# From Genetics to Histomolecular Characterization: An Insight into Colorectal Carcinogenesis in Lynch Syndrome

**DOI:** 10.3390/ijms22136767

**Published:** 2021-06-23

**Authors:** Martina Lepore Signorile, Vittoria Disciglio, Gabriella Di Carlo, Antonio Pisani, Cristiano Simone, Giuseppe Ingravallo

**Affiliations:** 1 Medical Genetics, National Institute for Gastroenterology, IRCCS “S. de Bellis” Research Hospital, 70013 Castellana Grotte, Italy; leporesignorile.labsimone@gmail.com (M.L.S.); disciglio.labsimone@gmail.com (V.D.); 2 Department of Emergency and Organ Transplantation, Section of Pathology, University of Bari Aldo Moro, 70124 Bari, Italy; gabry.dicarlo94@gmail.com; 3 Gastroenterology and Digestive Endoscopy Unit, National Institute for Gastroenterology, IRCCS “S. de Bellis” Research Hospital, 70013 Castellana Grotte, Italy; antonio.pisani@irccsdebellis.it; 4 Medical Genetics, Department of Biomedical Sciences and Human Oncology (DIMO), University of Bari Aldo Moro, 70124 Bari, Italy

**Keywords:** Lynch syndrome, CRC, early detection, MMR genes

## Abstract

Lynch syndrome is a hereditary cancer-predisposing syndrome caused by germline defects in DNA mismatch repair (MMR) genes such as *MLH1*, *MSH2*, *MSH6*, and *PMS2*. Carriers of pathogenic mutations in these genes have an increased lifetime risk of developing colorectal cancer (CRC) and other malignancies. Despite intensive surveillance, Lynch patients typically develop CRC after 10 years of follow-up, regardless of the screening interval. Recently, three different molecular models of colorectal carcinogenesis were identified in Lynch patients based on when MMR deficiency is acquired. In the first pathway, adenoma formation occurs in an MMR-proficient background, and carcinogenesis is characterized by *APC* and/or *KRAS* mutation and *IGF2*, *NEUROG1*, *CDK2A*, and/or *CRABP1* hypermethylation. In the second pathway, deficiency in the MMR pathway is an early event arising in macroscopically normal gut surface before adenoma formation. In the third pathway, which is associated with mutations in *CTNNB1* and/or *TP53*, the adenoma step is skipped, with fast and invasive tumor growth occurring in an MMR-deficient context. Here, we describe the association between molecular and histological features in these three routes of colorectal carcinogenesis in Lynch patients. The findings summarized in this review may guide the use of individualized surveillance guidelines based on a patient’s carcinogenesis subtype.

## 1. Introduction

Lynch syndrome (LS) is a hereditary disorder with an autosomal dominant transmission that primarily predisposes to colorectal and endometrial cancer, but is also associated with other extra-colonic malignancies, such as stomach, small bowel, pancreatic, bladder, prostate, and biliary tract cancers [1]. Hereditary colorectal cancer (CRC) in LS patients accounts for 3–5% of all CRC cases in adults [2]. LS carriers are born with germline mutations in DNA mismatch repair (MMR) genes, such as *MLH1*, *MSH2*, *MSH6*, and *PMS2*, or, more rarely, deletions in the 3′ end of the *EPCAM* gene that lead to hypermethylation of *MSH2* gene promoter. These mutations accelerate the inactivation of the second wild-type allele, accomplishing the classical Knudson’s two-hit hypothesis [3]. To date, heterozygous germline variants of other genes involved in the MMR pathway (*MSH3*, *MLH3*, and *PMS1*) have not been found, alone, to be causative of LS [4,5,6,7]. Inactivation of MMR genes leads to loss of MMR protein expression, which results in the accumulation of mutations in both coding and non-coding microsatellite regions (microsatellite instability; MSI) in tumor cells [8]. Indeed, microsatellite sequences are tracts of tandemly repeated DNA motifs ranging from one to six nucleotides in length, which are susceptible to accumulate mutations [9].

The selection of families for LS genetic testing is mainly based on personal and family cancer history using the Amsterdam criteria or the Bethesda guidelines [10,11]. Furthermore, in clinical practice, universal screening for LS based on MSI evaluation or MMR immunohistochemical (IHC) testing is recommended in order to identify patients who should be offered LS genetic testing [12]. MSI can be diagnosed by clinically useful tests: PCR or IHC analysis. Molecular testing is performed on DNA from fresh, frozen, or paraffin-embedded tumor tissue using a PCR-based assay. The highest specificity and sensitivity are reached using a panel of two or more polyA mononucleotide (BAT25, BAT26, NR-21, NR-22, NR-24, and NR-27) and three dinucleotide (D2S123, D5S346, and D17S250) markers. At least two of these different markers are needed to classify the tumor as MSI-high, whereas tumors with only one affected marker are considered MSI-low [9]. IHC is used to detect the expression of the four MMR proteins (MLH1, MSH2, MSH6, and PMS2), whose loss is highly concordant (>90%) with DNA-based assays. IHC positive staining is defined as unambiguous nuclear labeling in tumor cells, with staining intensity comparable to that of the internal control. A weak positive case is defined as an IHC stain that is visible as a nuclear label in tumor cells but whose intensity is lower than the internal control or only comparable to inert stromal cells. Loss of protein expression is defined as the complete absence of nuclear staining within tumor cells, with simultaneous positive labeling in non-neoplastic internal tissue [13].

**Figure 1:**
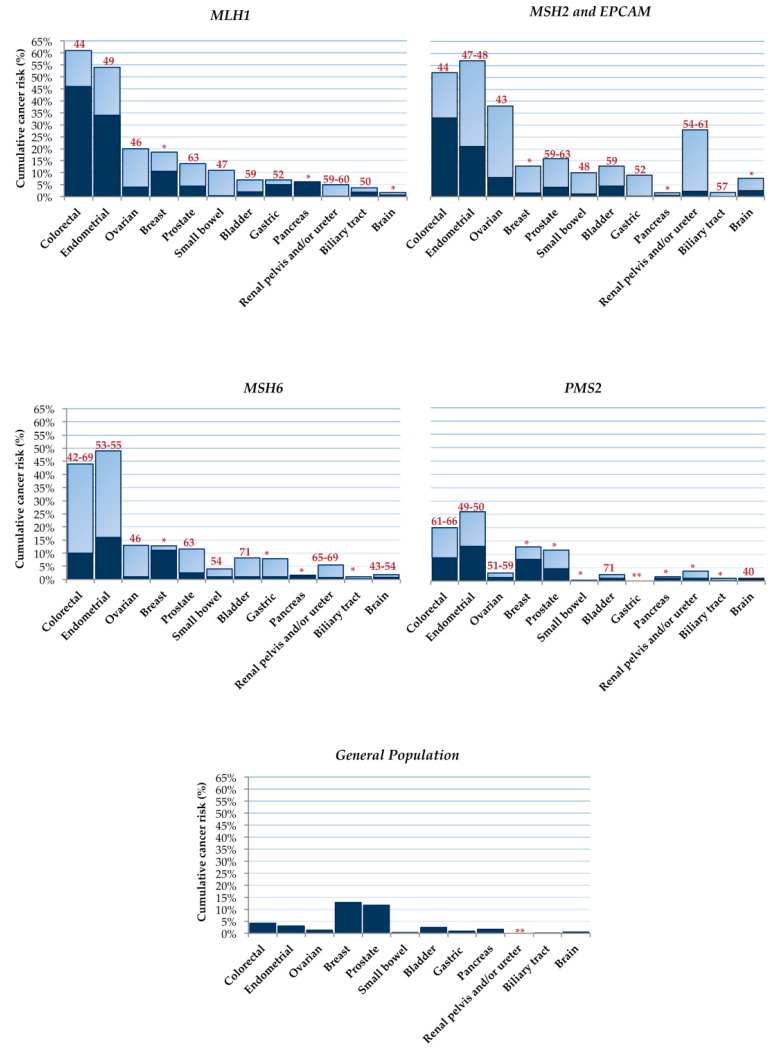
Cumulative risk of developing cancer in patients with LS according to the type of mismatch repair gene mutation by the age of 80. Dark and light blue bars indicate the lower and upper cumulative risk, respectively, for each cancer type. The red numbers above each bar represent the estimated mean age at presentation for each cancer type. Asterisks (*) and double asterisks (**) indicate that limited or no data are available for the mean age at presentation and the cumulative cancer risk, respectively.

Carriers of MMR pathogenic variants show an increased risk of developing a specific cancer type, and the relative cumulative risk depends on the underlying germline MMR defect. Several studies have been conducted with the aim of defining the cumulative risk of cancer in LS patients. The most recent National Comprehensive Cancer Network (NCCN) guidelines (Genetic/Familial High-Risk Assessment: Colorectal, Version: 1.2021; https://www.nccn.org/guidelines/category_2, accessed on 10 June 2021) for the management of familial CRC syndromes, including LS, report the cumulative cancer risk for specific DNA MMR gene mutations in LS carriers (Figure 1) [14,15,16,17,18,19,20,21,22,23,24,25,26,27,28,29].

*MLH1* and *MSH2* mutation carriers display a higher risk of cancer and an earlier age at presentation than *MSH6* and *PMS2* carriers. Endometrial cancer is the second most common cancer in women with LS, occurring in up to 54% of women with *MLH1* and up to 57% of women with *MSH2* or *EPCAM* mutations, while a lower risk (13–26%) is observed in *PMS2* mutation carriers [14,15,18,19,21,22]. Women with LS also have a higher risk of developing ovarian cancer [14,15,17,23]. The cumulative risk for urinary tract cancer in LS patients varies, ranging from less than 1% to 28%, with greater risk among carriers of *MSH2* mutations (2–28%) compared to carriers of *MLH1* (0.2–5%) or *MSH6* (0.7–5.5%) mutations [14,15,21,22,23,24,25,26]. Moreover, patients with LS are at higher risk for small bowel, stomach, hepatobiliary tract, prostate, and brain tumors [14,15,17,19,22,23,24,25,26,27,28,29]. Increased lifetime risk of pancreatic cancer has also been reported in LS patients [15], while the relative risk of breast cancer is not well established [15,17,22,23,25,26,28].

Currently, annual surveillance colonoscopy is recommended from the age of 25 years for *MLH1* and *MSH2* mutation carriers [18] and from the age of 30–35 for patients with deleterious mutations in *MSH6* and *PMS2* [30].

In the past, LS was often called hereditary nonpolyposis colorectal cancer (HNPCC) [3]. The term nonpolyposis CRC was meant to differentiate this condition, in which patients develop few early-onset adenomas (usually <10), from familial adenomatous polyposis (FAP), which is characterized by the presence of hundreds of adenomas. Moreover, accelerated adenoma-to-carcinoma progression has been reported in LS patients, with estimated polyp-to-cancer dwell times of 35 months compared with 10 to 15 years in sporadic cancer [31]. Colonoscopy is the most effective form of prevention in LS patients as it allows for the identification and removal of pre-invasive lesions and the diagnosis of early cancers in the absence of symptoms [18].

In fact, high-definition and quality colonoscopy enables gastroenterologists to recognize polypoid and non-polypoid lesions according to Paris classification and/or Kudo’s pit pattern classification. These procedures support the identification of characteristic alterations of the crypt morphology that are evocative of deep submucosal infiltration and thus help to assess whether radical surgical treatment will likely offer a better outcome than endoscopic removal. On the other side, the latter permits a preliminary histologic evaluation of the lesion to establish if polypectomy, mucosectomy, or submucosal dissection is indicated to obtain complete resection, without risk of nodal involvement [32,33].

Theoretically, a reduced incidence of CRC would be expected in patients undergoing more frequent colonoscopy, but emerging evidence supports increased CRC detection rates in post-colonoscopy LS patients. Indeed, in many cases, CRC becomes clinically manifest as an “interval” cancer, defined as a colon cancer that develops within 5 years of a complete colonoscopy and is therefore diagnosed between two screening colonoscopies [34]. Moreover, it is becoming evident that adenomas, the main precursors of CRC, can be missed during colonoscopy [2]. Indeed, in a meta-analysis of 15,000 tandem colonoscopies, miss rates were calculated to be 26% for adenomas and 9% for advanced adenomas, being particularly high for proximal advanced adenomas (14%), flat adenomas (34%), and in patients at high risk for CRC (33%) [35]. The precursor lesion of an LS-related CRC is an adenomatous polyp, which is often proximal and may frequently be non-polypoid rather than polypoid; besides, it frequently shows villous features, high-grade dysplasia, and a preponderance of tumor-infiltrating lymphocytes [2,30,36]. These types of CRC precursors may be difficult to recognize during colonoscopy. Due to the genetic predisposition of LS carriers, even small adenomas can be associated with accelerated progression along the adenoma–carcinoma sequence. Moreover, it is likely that in LS patients small adenomas do not remain dormant for many years as happens in the general population [37]. According to current evidence, surveillance approaches based on colonoscopy alone are still suboptimal in LS carriers, regardless of the screening interval [38]. Although no other options are currently available for effective non-invasive screening, novel modalities are emerging in order to optimize early detection of CRC in LS patients, including the use of next-generation sequencing (NGS) to complement colonoscopy [39].

**Figure 2:**
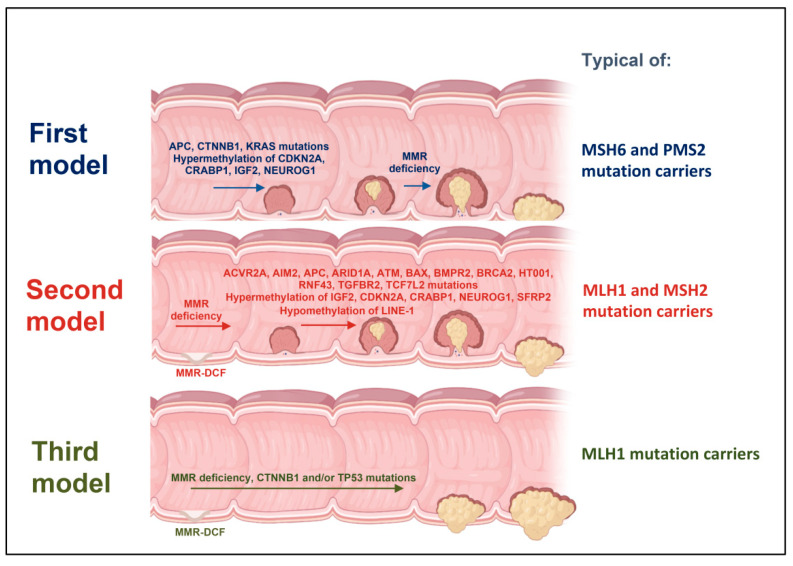
Three different models of colorectal carcinogenesis in Lynch syndrome patients.

Interestingly, CRC patients with LS show distinctive phenotypic hallmarks, such as preferential tumor localization in the right-sided colon, presence of multiple synchronous and metachronous CRCs, and poorly differentiated tumors [3]. To date, three different types of colorectal carcinogenesis have been characterized in LS patients based on the time of MMR deficiency onset. These patterns are characterized by different mutation spectra and histological features [40]. In the first pattern, tumors arise from polypoid lesions within an MMR-proficient background. However, most commonly, MMR deficiency is an early event in tumor formation and promotes the development of precursor lesions termed MMR-deficient crypt foci (MMR-DCF), which can progress either through a non-polypoid adenomatous phase (second type of carcinogenesis) or lead directly to invasive cancer (third type of carcinogenesis), which is why colonoscopy alone does not currently seem to be sufficient for early diagnosis [40] (Figure 2).

## 2. First Model of LS Colorectal Carcinogenesis: Adenoma Growth in an MMR-Proficient Background

Classical colorectal carcinogenesis follows the adenoma–carcinoma sequence, with MMR deficiency arising after adenoma development [41]. Indeed, for decades MMR deficiency was considered to be a secondary event in LS carcinogenesis. This idea was sustained by several studies noticing that adenomas retained the expression of MMR proteins [8]. However, it has recently been demonstrated that only up to 25% of all adenomas in LS patients are MMR proficient [42]. The existence of MMR-proficient adenomas supports the idea that tumor formation is possible even in the absence of the second hit required to inactivate the wild-type allele of an MMR gene. This model of carcinogenesis presumes the existence of other somatic events to initiate malignant transformation [8]. *APC* and *KRAS* mutations may represent the starting events and are believed to enhance tumorigenesis when MMR gene expression is still intact. Loss or inactivation of the *APC* gene on chromosome 5q drives the growth of small adenomas with a hypomethylated genome by promoting clonal expansion. Furthermore, almost all *KRAS* mutations appearing before MMR deficiency in LS patients are alterations involving specific sequences, such as *KRAS* G12V and *KRAS* A146T [8].

Epigenetic changes are defined as stable and hereditable alterations in gene expression and cell functions without changes in the original DNA sequence. Epigenetics plays a role in CRC in two different ways: on the one hand, the genome of the bulk tumor appears hypomethylated compared to normal colonic epithelia; on the other hand, there are particular regions, such as CpG islands, which are normally unmethylated, that appear hypermethylated. CpG islands are associated with the promoters of genes involved in several cell functions, including cell cycle control (*CDKN2A*), DNA repair (*MLH1*), and apoptosis (*DAPK*) [43]. Widespread DNA methylation at CpG sites in promoter regions [44] can be a putative “second hit” in LS carriers [45] and likely precedes MSI, representing an early event in tumor development. Indeed, aberrant CpG island methylation affecting several tumor suppressor genes leads to a CpG island methylator phenotype (CIMP) [45]. Moreover, Maki-Nevala and colleagues found higher methylation levels in four CIMP marker genes, namely *NEUROG1, CDKN2A, IGF2,* and *CRABP1*, in MMR-proficient adenomas compared to normal mucosa [45,46].

All these markers were identified hypermethylated also in these adenomas when MMR deficiency is acquired [8].

The *NEUROG1* gene, located on chromosome 5 (5q23–q31), encodes for a transcription factor that binds to E-box elements. Methylation analysis of *NEUROG1* in CRC tumors showed progressive hypermethylation associated with neoplastic development; indeed, increasing methylation levels were found from normal to tumor mucosa [47]. Currently, *NEUROG1* is considered a potential diagnostic marker for early CRC since its methylation status can be detected in patient sera in a non-invasive way by population-wide screening for colorectal neoplasia, which is especially useful for people who refuse colonoscopy [48].

*CDKN2A* is an important tumor suppressor in CRC. Methylation of its promoter leads to *CDKN2A* gene silencing [49] and ultimately promotes uncontrolled cell proliferation [50]. Several studies evaluated the association between *CDKN2A* hypermethylation and shorter CRC patient survival, suggesting its role as an independent prognostic factor that might predict invasion and metastasis [51].

*IGF2* encodes a protein that plays a major role in growth and development after birth. It is frequently altered in cancer and is involved in neoplastic proliferation [52]. LS patients with CRC show a higher amount of hypermethylated *IGF2* in adenoma and adenocarcinoma tissue compared to normal colon mucosa [45]. Interestingly, *IGF2* can be used as a prognostic marker since its methylation status can be screened by epigenetic blood testing in order to identify early in life LS carriers that are highly susceptible to developing CRC [53].

CRABP1 belongs to a family of fatty acid-binding proteins and is associated with a poor prognosis in several cancers. A recent study showed significantly elevated methylation levels of *CRABP1* in MMR-proficient adenomas of LS patients [8].

From a histological point of view, the first model of LS carcinogenesis is characterized by the development of polypoid precursor lesions frequently found in the right side of the colon [36,54,55].

Riijcken and colleagues reported that adenomas in the right side of the colon are more prone to malignant conversion than left-sided adenomas [36] and have a short dwell time [55]. Consistently, Edelstein and colleagues estimated that adenoma dwell time in LS patients is considerably shorter than in patients with sporadic CRC [31]. Several studies reported a low incidence of serrated lesions in LS carriers, with the majority of adenomas appearing as conventional adenomas and/or hyperplastic polyps [56]. Moreover, most of them (about 80%) have a flat morphology, which is frequently missed during conventional endoscopic exams [2]. Other histological signs are the presence of differentiated mucinous cells and seal ring cells, a medullary growth model associated with a marked lymphocytic peritumoral inflammation that recalls the characteristics of the so-called ‘Crohn’s reaction’ [13].

In this type of colorectal carcinogenesis, the adenoma–carcinoma sequence is extremely accelerated [55,57], so that even colonoscopies performed annually may not be effective [58].

This first model of LS colorectal carcinogenesis is probably the most frequent pathway of tumor initiation in *MSH6* and *PMS2* mutation carriers. This finding is supported by the fact that low-grade adenomas in patients with *MSH6* and *PMS2* alterations are frequently microsatellite stable. Furthermore, Engel et al. reported a significant molecular signature in which *MSH6* mutation carriers are associated with low frequency of *CTNNB1* mutations and high frequency of *APC* mutations, suggesting that in these patients the onset of MMR deficiency only occurs after adenoma formation. Besides, *MSH6* mutation carriers appear to be at low risk of cancer, probably because the isolated loss of *MSH6* gene function does not completely abrogate MMR activity due to the overlapping functions of *MSH3* [59]. Indeed, patients bearing alterations in *MSH6* or *PMS2* benefit more from current colonoscopy surveillance programs than *MLH1* or *MSH2* mutation carriers due to the different type of tumorigenesis involved [60] and have been reported to have a lower risk of developing CRC along with a later age at presentation [61]. Interestingly, the few *MLH1* and *MSH2* mutation carriers exhibiting the first pattern of LS carcinogenesis are susceptible to develop somatic mutations in *CTNNB1* and *APC* genes, respectively, before the growth of adenomas [41].

Importantly, the first model of colorectal carcinogenesis in LS patients is characterized by the growth of adenomas as tumor precursor lesions in an MMR-proficient background, but Sekine and colleagues suggest that MMR deficiency occurs in adenomas before the progression to carcinomas, emphasizing the relevance of MMR impairment during LS carcinogenesis [62].

## 3. Second Model of LS Colorectal Carcinogenesis: MMR-DCF Leading to Adenoma Formation and Transition to Carcinoma

In recent years, the classic view of LS as an “accelerating” disease has been challenged, especially by the identification of MMR-DCF, which are colon crypts showing mostly normal histological features but already lacking MMR protein expression. This observation has suggested that MMR-deficient CRC in LS patients could also begin from such MMR-DCF [40]. Indeed, several studies have shown that about 75% of all adenomas in LS patients are MMR deficient [40,41].

From a histological point of view, MMR deficiency in LS patients can be detected heterogeneously in dysplastic crypts when it occurs in an already existing adenoma, while it can be observed consistently in adjacent and non-dysplastic MMR-DCF when it precedes adenoma formation [40]. This latter lesion type is unique to LS patients’ CRCs and has not been detected in sporadic CRC [61,63]. The occurrence of thousands of MMR-DCF, approximately 1 MMR-DCF per cm^2^ of mucosa, has been observed in phenotypically normal intestinal mucosa [61]. These lesions can be considered tumor precursors in LS patients and are difficult to detect by colonoscopy at a pre-invasive stage [41]. Importantly, most of these lesions do not seem to progress to malignancy, as suggested by the discrepancy between the large number of MMR-DCF and the small number of adenomas or carcinomas observed in LS carriers [61].Histologically, MMR-DCF are almost indistinguishable from normal colonic crypts. Slight differences are the nuclear enlargement of cells at the bottom of the crypt and features of neoplastic growth such as aberrant branching and typical crypt fission. Moreover, MMR-DCF appear as non-elevated lesions without widened luminal openings [61]. Despite these differences, MMR-DCF retain the potential to migrate and mature along the crypt–villus axis [42], and Ki-67 staining showed a physiological proliferation pattern similar to that observed in normal mucosa [61]. For this reason, MMR-DCF escape routine histological detection with methylene blue staining. Besides, morphological evaluation of MMR-DCF-adjacent mucosal areas did not reveal marks of altered immune infiltration [63]. This type of lesion is significantly associated with patients’ age at the time of tumor resection and with cancer location, being more frequent in patients with distal colorectal tumors [63]. This model of LS carcinogenesis is initiated by non-polypoid precursor lesions that can directly give rise to localized adenocarcinomas.

All tumors of LS patients displaying this type of colorectal carcinogenesis show MSI [64], which is characterized by instability in coding and non-coding short repeat microsatellite sequences caused by mutations in MMR genes [65]. These mutations can lead to reading frame shifts resulting in the inactivation of key tumor suppressor genes with growth-related functions [66]. Indeed, the above histological features are associated with molecular signatures typically identified upon mutations in major tumor suppressor genes, such as *TGFBR2* [42]. *TGFBR2* encodes for a type II TGF-β receptor that can activate the TGF-β pathway by specific ligand binding [67]. The TGF-β signaling pathway is involved in the inhibition of cell proliferation and the induction of apoptosis [68]. *TGFBR2* gene sequence architecture is prone to replication errors because it contains several repeated DNA motifs [69], including ten adenosine residues that are frequently targeted by MMR gene inactivation [63]. Mutations in *TGFBR2* were found in 80% of early colorectal adenomas with high microsatellite instability [70]. This finding suggests that *TGFBR2* mutations occur early after bi-allelic inactivation of MMR genes [61], but other studies revealed that *TGFBR2* mutations are also involved in neoplastic progression since this gene is frequently increased in typical advanced lesions such as poly-cryptic MMR-DCF [63]. Conversely, mono-cryptic MMR-DCF frequently show mutations in the microsatellite coding regions of *HT001*, *AIM2*, and *BAX* genes [63]. Interestingly, Pinheiro and colleagues detected the co-occurrence of *TGFBR2*, *ACVR2A*, and *BMPR2* mutations, indicating that loss of one of these receptors is likely insufficient for complete TGF signaling disruption [71]. Furthermore, the molecular signature associated with the second model of LS colorectal carcinogenesis suggests the involvement of the WNT signaling pathway. Of note, low mutation frequency has been detected in core genes of this pathway, such as *AXIN1*, *AXIN2*, *PTEN*, and *CTNNB1* [72]. Conversely, this signature is characterized by *RNF43* mutations, mostly by frameshift affecting mononucleotide repeats [72]. *RNF43* is a ubiquitin ligase of Frizzled cell surface receptors and acts as a negative regulator of the WNT pathway, which is frequently altered during CRC carcinogenesis [73]. Furthermore, despite the low frequency of *APC* mutations occurring after MMR deficiency onset, almost all of these mutations are insertions or deletions involving specific repeat sequences, such as an A5-repeat at codon 1455, an AG5-repeat at codon 1465, and an A6-repeat at codon 1554 [62]. Noteworthy, *RNF43* and *APC* mutations, which both lead to WNT pathway activation and are usually mutually exclusive, often co-exist in LS patients, both in adenomas and adenocarcinomas [62]. The importance of the WNT pathway in LS carcinogenesis is also highlighted by the common transcriptional silencing of *SFRP2*, another WNT signaling antagonist. Indeed, hypermethylation of the *SFRP2* promoter induces uncontrolled cell proliferation [45]. Moreover, several studies reported a high mutation frequency of *TCF7L2*, which encodes a component of the WNT signaling pathway and is associated with an increased risk of CRC. Indeed, up to 60% of LS patients carry mutations in *TCF7L2* [71,74,75].

LS patients with the second type of colorectal carcinogenesis also display a high mutation rate in genes involved in DNA damage response signaling, such as *ARID1A*, *ATM*, and *BRCA2*, which play a role in homologous recombination and double-strand break repair [72].

Interestingly, this model of LS colorectal carcinogenesis is also associated with decreased methylation levels of LINE-1 (long interspersed nuclear elements) [63]. This epigenetic alteration may predispose cells to chromosomal rearrangements, resulting in increased mutation rates [76].

Moreover, MMR-deficient adenomas with a high grade of dysplasia revealed hypermethylation in four other genes, *IGF2, CRABP1, NEUROG1,* and *CDKN2A* [8].

The second model of LS colorectal carcinogenesis is characterized by the early acquisition of MMR deficiency, which precedes adenoma formation. This pathway is probably the most frequent type of carcinogenesis in *MLH1* and *MSH2* mutation carriers and does not show substantial differences in MMR-DCF frequency or with patient gender. Moreover, it has been reported that *MSH6* and *PMS2* mutation carriers can benefit more from colonoscopy surveillance since MMR-DCF are both less common and less likely to progress along the adenoma–carcinoma sequence [77].

## 4. Third Model of LS Colorectal Carcinogenesis: MMR-DCF Showing Direct Transition to Carcinoma

About 10% of LS-associated cancers are prone to skip the adenoma step of the classical adenoma–carcinoma sequence in the carcinogenesis process [58]. This quickly leads to an invasive phenotype presumably arising from MMR-DCF through somatic mutations in genes such as *CTNNB1* and *TP53* [41]. Since MMR-DCF can grow under an intact mucosal surface, these types of lesions frequently escape colonoscopic detection during recommended surveillance [40], directly evolving into manifest cancer without a macroscopically visible non-invasive precursor [42].

Indeed, unlike *APC* mutations, *CTNNB1* mutations mostly occur in non-polypous CRCs lacking the adenomatous precursor stage [40]. *CTNNB1* mutations are commonly observed in this type of colorectal carcinogenesis in LS patients and require at least one additional pre-existing alteration to exert their oncogenic effects and drive tumorigenesis in colonic mucosa [58]. The *CTNNB1* mutations observed in LS cancer are frequently an amino acid substitution at codon 41 (c.121A > G) or other point mutations at codon 45, such as c.133T > C or c.134C > T [77]. In addition, *CTNNB1* in-frame deletions have also been described, including c.133_135del or other deletions encompassing c.133 that result in the removal of the serine residue in position 45 (S45) [58]. S45 is a phosphorylation site for casein kinase-1 and is involved in the regulation of β-catenin stability; indeed, S45 mutations result in stronger activation of WNT/β-catenin signals and are frequently associated with malignant transformation [78].

In the two patterns of LS colorectal carcinogenesis described above, polypous regions are observed adjacent to the invasive margin of the tumors, while most cancers associated with the third type of LS colorectal carcinogenesis lack these histological features. Since MMR-DCF do not always evolve spontaneously into a neoplastic lesion, Ahadova and colleagues suggested that mutations in *CTNNB1* and/or *TP53* may be involved in the malignant conversion of these foci [58]. Despite *TP53* mutations are believed to be uncommon in LS patients, Maki-Nevala and colleagues reported that up to 33% of LS adenomas showed mutations in the *TP53* gene [8].

This third model of LS colorectal carcinogenesis is characterized by fast tumor growth with invasive features in an MMR-deficient context. This pattern is frequently associated with *MLH1* mutations [41], while it is not observed in *PMS2* mutation carriers, which may explain the reduced risk of CRC in these patients under surveillance programs [34].

## 5. Conclusions and Future Directions

Since LS patients do not seem to fully benefit from current surveillance strategies, researchers and clinicians are in the search for novel diagnostic approaches to prevent CRC development.

In order to optimize current surveillance programs for LS carriers, more sophisticated endoscopic techniques, such as chromoendoscopy, virtual chromoendoscopy as narrow-band imaging (NBI), could be implemented to improve the detection of adenomas compared to normal colonoscopy. These approaches would significantly benefit LS patients with the first and second models of colorectal carcinogenesis described above [34,79,80]. Colonic chromoendoscopy takes advantage of the topical application of stains such as indigo carmine, a deep-blue contrast dye, in order to improve mucosal contrast [81]. This technique allowed the detection of up to twice the number of adenomas compared to normal colonoscopy and identified a higher proportion of patients with at least one adenoma [82,83,84,85]. Importantly, no significant side effects were observed [82]. Other studies demonstrated that the use of colonoscopy with NBI also doubled the number of adenomas detected in LS patients [86].

In the future, artificial intelligence applied to colonoscopy (computer-aided diagnosis or CAD) could help to increase the adenoma detection rate through enhanced evaluation of superficial (epithelial and vascular) irregularities by using dedicated software to reduce missed adenomas [87].

**Figure 3:**
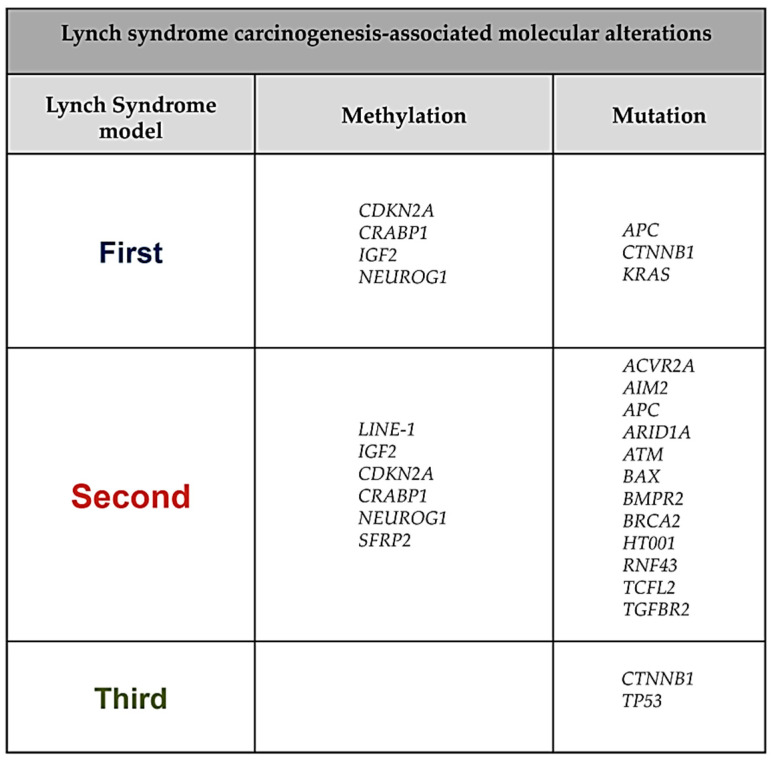
List of potential molecular markers that could be analyzed non-invasively for methylation or mutational status by testing distant media (stool or blood).

LS patients could additionally benefit from novel screening modalities based on NGS, which are extremely informative in guiding surveillance [88]. Screening of distant media such as stool or blood could be incorporated in current surveillance programs of LS patients to detect genetic and epigenetic cancer-specific alterations. Indeed, the investigation of DNA markers methylation status characteristic of LS tumorigenesis could help to detect the presence of precursor lesions and CRC. Ballester and colleagues searched for novel markers to be used in a test that is already clinically available. These authors found that *OPLAH* alone and/or a combination of a few methylated DNA markers (*ARHGEF4, LRRC4, ANTXR1, PITX1*) can discriminate adenomas and CRC in LS patients [39]. All these DNA markers represent aberrantly hypermethylated sequences; indeed, the hypomethylation status is more difficult to recognize in distant media. Interestingly, aberrant hypermethylation of *ALKBH5*, a gene involved in DNA damage signaling, has been recognized as a unique sign in LS neoplasms compared to CRC sporadic neoplasms [39]. Ideally, a specific large panel should be created including these and other DNA markers identified in other studies, such as *NEUROG1, CDKN2A, IGF2*, and *CRABP1*. In the personalized medicine era, NGS-targeted gene panels comprising top-performing markers as cancer-specific mutations (*TGFBR2, RNF43, ARID1A, ATM, BRCA2, CTNNB1*, and others) may optimize early detection of CRC precursors and increase compliance in these high-risk patients (Figure 3).

Interestingly, the results gathered from these analyses can be used not only for diagnostic procedures but also for therapeutic approaches in LS patients. Indeed, ovarian, breast, pancreas, and prostate cancer patients with somatic *BRCA* mutations can be currently treated with poly(ADP-ribose) polymerase (PARP) inhibitors such as olaparib, rucaparib, niraparib, and talazoparib [89,90,91,92,93]. The increased frequency of somatic *BRCA2* mutations documented in MMR-deficient LS carriers suggests the potential application of PARP inhibition therapy [72]. Moreover, growing evidence indicates that alteration of other major homologous recombination repair (HRR) proteins, such as ATM, may induce a response to PARP inhibitors in patients affected by multiple solid malignancies, such as prostate cancer and triple-negative breast cancer [94,95]. Indeed, various preclinical studies have shown that CRC cell lines carrying *ATM* mutations exhibit increased sensitivity to olaparib [96]. These data support the development of HRR inhibition therapy as a promising anticancer strategy also in LS patients.

Larger randomized trials are needed in order to apply these novel screening modalities in current clinical settings. These studies are necessary to provide sufficient statistical power to validate the effectiveness of these novel markers of colorectal carcinogenesis in LS patients.

Since no survival gain was observed in LS patients undergoing more frequent colonoscopies, the findings described above support the use of an individualized diagnostic approach based not only on the MMR mutation carried by the patient but also on the pattern of histological and molecular carcinogenesis. In this light, in addition to standard colonoscopy, LS patient screening programs should include sophisticated endoscopic techniques combined with non-invasive approaches based on NGS to analyze specific markers in distant media.
